# The impact of proton-pump inhibitors administered with tyrosine kinase inhibitors in patients with metastatic renal cell carcinoma

**DOI:** 10.1097/CAD.0000000000001356

**Published:** 2022-10-22

**Authors:** Sebastiano Buti, Chiara Tommasi, Giulia Scartabellati, Ugo De Giorgi, Nicole Brighi, Sara Elena Rebuzzi, Silvia Puglisi, Orazio Caffo, Stefania Kinspergher, Alessia Mennitto, Carlo Cattrini, Matteo Santoni, Elena Verzoni, Alessandro Rametta, Marco Stellato, Andrea Malgeri, Giandomenico Roviello, Massimo de Filippo, Alessio Cortellini, Melissa Bersanelli

**Affiliations:** a Medical Oncology Unit, University Hospital of Parma; b Department of Medicine and Surgery, University of Parma; c Gruppo Oncologico Italiano di Ricerca Clinica (GOIRC), Parma; d Department of Medical Oncology, Istituto Scientifico Romagnolo per lo Studio e la Cura dei Tumori (IRST) IRCCS, Meldola; e Medical Oncology Unit, Ospedale San Paolo, Savona; f Department of Internal Medicine and Medical Specialties (Di.M.I.), University of Genova; g Medical Oncology Unit 1, IRCCS Ospedale Policlinico San Martino, Genova; h Department of Medical Oncology, Santa Chiara Hospital, Trento; i Division of Oncology, University Hospital ‘Maggiore della Carità’, Novara; j Department of Medical Oncology, University of Marche, University Hospital Ospedali Riuniti, Ancona; k Department of Medical Oncology, Fondazione IRCCS Istituto Nazionale Tumori, Milan; l Department of Medical Oncology, Università Campus Bio-Medico di Roma, Rome; m Department of Health Sciences, University of Florence, Florence; n Radiology Unit, University Hospital of Parma, Parma, Italy; o Department of Surgery and Cancer, Imperial College London, London, UK

**Keywords:** cabozantinib, interaction, metastatic renal cell carcinoma, pazopanib, proton-pump inhibitors, tyrosin kinases inhibitors

## Abstract

Tyrosine kinase inhibitors (TKIs) are the backbone of the systemic treatment for patients with metastatic renal cell carcinoma (mRCC). TKIs such as pazopanib and cabozantinib can interact with other drugs concomitantly administered, particularly with proton-pump inhibitors (PPIs), possibly impacting the effectiveness of the anticancer treatment and patients outcome. Few data are available about this interaction. We conducted a multicenter retrospective observational data collection of patients with mRCC treated with pazopanib or cabozantinib between January 2012 and December 2020 in nine Italian centers. Univariate and multivariate analyses were performed. The aim was to describe the impact of baseline concomitant PPIs on the outcome of patients to pazopanib and cabozantinib in terms of response, progression-free survival (PFS) and overall survival (OS), toxicity, and treatment compliance. The use of PPI in our study population (301 patients) significantly influenced the effectiveness of TKIs with worse PFS (16.3 vs. 9.9 months; *P* < 0.001) and OS (30.6 vs. 18.4 months; *P* = 0.013) in patients taking PPI at TKI initiation. This detrimental effect was maintained both in the pazopanib and cabozantinib groups. The use of PPI influenced the toxicity and TKI treatment compliance with a reduction of dose or schedule modifications, and treatment interruptions in the population taking PPIs. Our study demonstrates that the use of PPIs can significantly influence the outcome and compliance of patients with mRCC to TKI treatment, suggesting the importance of a more careful selection of patients who need a gastroprotective therapy, avoiding indiscriminate use of PPIs.

## Introduction

Drug–drug interactions (DDIs) represent a crucial issue in oncology. Most advanced solid cancers are subtended by risk factors common to other relevant comorbidities, often implying a significant number of concomitant medications. In addition, the overuse of compounds able to impact the gut microbiota, namely antibiotics and proton-pump inhibitors (PPIs), is recently increasing beyond their proper indications, leading to an unmotivated overuse of such drugs. The risk of their direct and indirect interactions with anticancer drugs is potentially extremely high in terms of bioavailability, pharmacokinetics, and immunological interference, the latter possibly mediated by microbiota. Consequently, the effectiveness of anticancer compounds is likely to be altered by such concomitant medications [1].

When considering PPIs, both their mechanism of action, blocking gastric acid secretion, and their metabolism, mediated by hepatic cytochrome P450 (CYP) enzymes, can contribute to the likeliness of interactions with anticancer drugs. In particular, more than the pharmacokinetic impact based on the saturation of the cytochrome P450 metabolic pathways by multiple concomitant mediations, the main expected impact of PPIs is likely exerted by the reduction in the gastric pH and the consequent alterations, both in terms of drug absorption and gut microbiota composition. Two main classes of anticancer drugs could be impacted in their efficacy by such latter mechanisms: orally administered tyrosine kinase inhibitors (TKIs) and immune checkpoint inhibitors [2].

On one hand, the potential interactions with immunotherapeutic compounds may be even more complex, involving immune-mediated responses and leading to controversial clinical evidence between a negative or a positive impact on the outcome of patients [3,4]. On the other hand, considering the cruciality of TKIs bioavailability for their effectiveness and the strict correlation between drug efficacy and exposure, a predominance of direct interactions can be hypothesized between PPIs and TKIs.

The first step potentially impacted by PPIs is TKI solubility, which is pH-dependent: when the intragastric pH is elevated, the nonionized form of the TKI drug becomes prevalent over the ionized ones, and the bioavailability decreases. Nevertheless, the intragastric pH is not elevated over the full 24-h range during PPI therapy: thus, the bioavailability can significantly vary based on the timing of the respective intake of the two compounds [5]. Moreover, additional interference can be exerted by confounding factors, such as food intake and behavioral habits: for example, some authors demonstrated that the assumption of TKIs together with acidic beverages can cancel their DDI with PPIs [6]. Also, food intake enhances TKI bioavailability by elevating intragastric pH and drug absorption [7].

The pharmacokinetic interference represents the second step potentially contributing to DDI between PPIs and TKIs. Nevertheless, on the one hand, the principal CYP enzyme, CYP3A4, is implicated in the metabolism of almost all TKIs, whereas PPIs are metabolized via CYP2C19, having the dominant role besides other CYP enzymes [2]. Therefore, a pure pharmacokinetic interaction seems likely not as clinically relevant.

Finally, an indirect influence mediated by microbiota alterations, possibly not only on the efficacy outcome but even in terms of TKI tolerability, is largely presumable as the third step contributing to this DDI, especially considering the increasing evidence about the potential of PPIs in modulating the gut microbiota composition. The gastric pH modulation may differently select the bacterial species able to survive throughout the transit to the gut, and the balance of the microbiota can be heavily impacted by PPIs, as demonstrated by the reports about an increased risk of *Clostridium difficile* infection or colonization by multidrug-resistant Enterobacterales and vancomycin-resistant enterococci with long-term PPI use [8,9]. On the other hand, the influence of microbiota on TKI therapy outcome recently emerged, as suggested by the evidence that fecal microbiota transplantation allows treating diarrhea induced by TKI in patients with metastatic renal cell carcinoma (mRCC) [10].

Considering such variables, the objective of discriminating the selective impact of PPIs on the anticancer TKI treatment outcome requires analyses on vast and homogeneous patient populations. For this reason, with the aim to describe the potential clinical impact of the DDI between PPIs and TKIs, we evaluated a population of patients diagnosed with mRCC treated with cabozantinib or pazopanib, orally administered anti-VEGF TKIs with a once-per-day intake.

## Methods

We conducted a multicenter retrospective observational data collection of patients with mRCC treated with pazopanib or cabozantinib between January 2012 and December 2020 in nine Italian centers involved in the study. The objective of the study was to assess the impact of baseline PPIs on the outcome of patients to anticancer therapy in terms of response, progression-free survival (PFS), overall survival (OS), toxicity, and treatment compliance.

### Patients

Patients with a histological diagnosis of RCC treated with pazopanib or cabozantinib in the advanced setting were included in the study. Any line of treatment was allowed.

The prognostic group for each patient was evaluated at baseline using the International mRCC Database Consortium (IMDC) criteria [11]. Information about the baseline Eastern Cooperative Oncology Group Performance Status (ECOG PS), metastatic sites, grading of toxicity developed during the TKI treatment, treatment compliance outcome, and biochemical parameters were also required. In addition, we collected baseline data about concomitant medications used by the patients, particularly PPIs, and not interrupted at the time of TKI initiation.

The approval by the ethics committee of the coordinating center and informed consent for each patient were obtained.

### Statistics

Descriptive statistics were used to report patients’ characteristics. The radiological assessment was performed according to the local practice of centers about every 3 months, usually using Response Evaluation Criteria in Solid Tumours.

We defined as ‘responders’ patients with complete response (CR), partial response (PR), or stable disease (SD) as the best response; patients with progressive disease (PD) as the best response or with response not evaluable (NE) due to clinically PD were defined as ‘nonresponders’.

PFS was calculated from the start of therapy with pazopanib or cabozantinib to the disease progression or death, whichever occurred first. OS was measured from the start of treatment with pazopanib or cabozantinib to death. Patients without progression or death at the last follow-up were considered as censored.

PFS and OS were estimated using the Kaplan–Meier method with Rothman’s 95% confidence intervals (CIs) and compared using the log-rank test. The median follow-up was calculated with the reverse Kaplan–Meier method. Univariate and multivariate analyses were performed by using Cox proportional hazard models. The Chi-square test was used to compare categorical endpoints. Significance levels were set at a value of 0.05, and all *P*-values were two-sided.

Adverse events (AEs) related to TKI treatment were graded according to the Common Terminology Criteria for AE.

The compliance of patients was evaluated in terms of dose reductions, temporary interruptions, and schedule modifications of TKI treatment.

We planned the analyses for the overall patient population and each treatment group (pazopanib or cabozantinib).

The PASW software (Predictive Analytics Software, IBM Corp., Released 2012. IBM SPSS Statistics for Windows, Version 28.0, IBM Corp., Armonk, New York, USA) was used for the analyses.

## Results

### Patient characteristics

We enrolled 301 patients. The characteristics of the overall population and according to the TKI group are shown in Table 1: 179 patients (59%) were treated with pazopanib and 122 (41%) with cabozantinib.

**Table 1:** Characteristics of patients, overall and by tyrosin kinase inhibitor use

Number of patients (%)	Overall	Pazopanib group	Cabozantinib group
	301 (100%)	179 (59%)	122 (41%)
Median age (range)	68 (36–89)	70 (42–89)	65 (36–85)
Sex (%)			
Male	206 (68.4)	126 (70.4)	80 (65.6)
Female	95 (31.6)	52 (29.4)	42 (34.4)
Histology (%)			
Clear cell	250 (83.1)	152 (84.9)	98 (80.3)
Papillary	24 (8.0)	11 (6.1)	13 (10.7)
Chromophobe	8 (2.7)	5 (2.8)	3 (2.5)
Other	19 (6.3)	11 (6.1)	8 (6.6)
IMDC score (%)			
Good	103 (34.2)	65 (36.3)	38 (31.1)
Intermediate	159 (52.8)	92 (51.4)	67 (54.9)
Poor	39 (13.0)	22 (12.3)	17 (13.9)
ECOG PS (%)			
0	183 (60.8)	10 (61.5)	73 (59.8)
1	102 (33.9)	61 (34.1)	41 (33.6)
2–3	16 (5.4)	8 (4.5)	8 (6.5)
NLR (%)			
<3	183 (60.8)	80 (44.7)	43 (35.2)
≥3	102 (33.9)	75 (41.9)	65 (53.3)
NA	38 (12.6)	24 (13.4)	14 (11.5)
Nephrectomy (%)			
Yes	256 (85)	149 (83.2)	107 (87.7)
No	45 (15)	30 (16.8)	15 (12.3)
Median number of metastatic sites (range)	2 (1–8)	2 (1–6)	3 (1–8)
Sites of metastasis (%)			
Lung	194 (64.5)	116 (64.8)	78 (63.9)
Liver	58 (19.3)	29 (16.2)	29 (23.8)
Nodes	126 (41.9)	58 (32.4)	68 (55.7)
Bone	112 (37.2)	53 (29.6)	59 (48.4)
Glands	58 (19.3)	30 (33.5)	28 (23.0)
Other	114 (37.9)	60 (33.5)	54 (44.3)
Use of PPI (%)			
Yes	132 (43.9)	69 (38.5)	63 (51.6)
No	169 (56.1)	110 (61.5)	59 (48.4)
Line of treatment (%)			
1st	192 (63.8)	175 (97.8)	17 (13.9)
2nd	54 (17.9)	3 (1.0)	51 (41.8)
≥3rd	55 (18.3)	1 (0.2)	54 (44.2)

ECOG PS, Eastern Cooperative Oncology Group Performance Status; IMDC, International mRCC Database Consortium criteria; NA, not available; NLR, neutrophil-to-lynphocyte ratio; PPI, proton-pump inhibitors.

The median age was 68 years, with a male:female ratio of 2:1. Most patients (53%) had an intermediate prognostic score according to IMDC criteria. The same percentage was consistent in both TKI groups. Patients received pazopanib as the first-line treatment in 97% of cases; cabozantinib was used mainly beyond the first-line (42% of patients received it as the second-line treatment and 44% after the second-line therapy). The other characteristics were similar in both groups.

The median number of metastatic sites in the overall population was two. Lung, lymph nodes, and bone were involved respectively in 64%, 42%, and 37% of cases.

Overall, 44% of patients were taking a PPI at the time of PPI initiation: 39% in the pazopanib group and 52% in the cabozantinib group.

### Oncological outcome

#### Progression-free survival

**Fig. 1:**
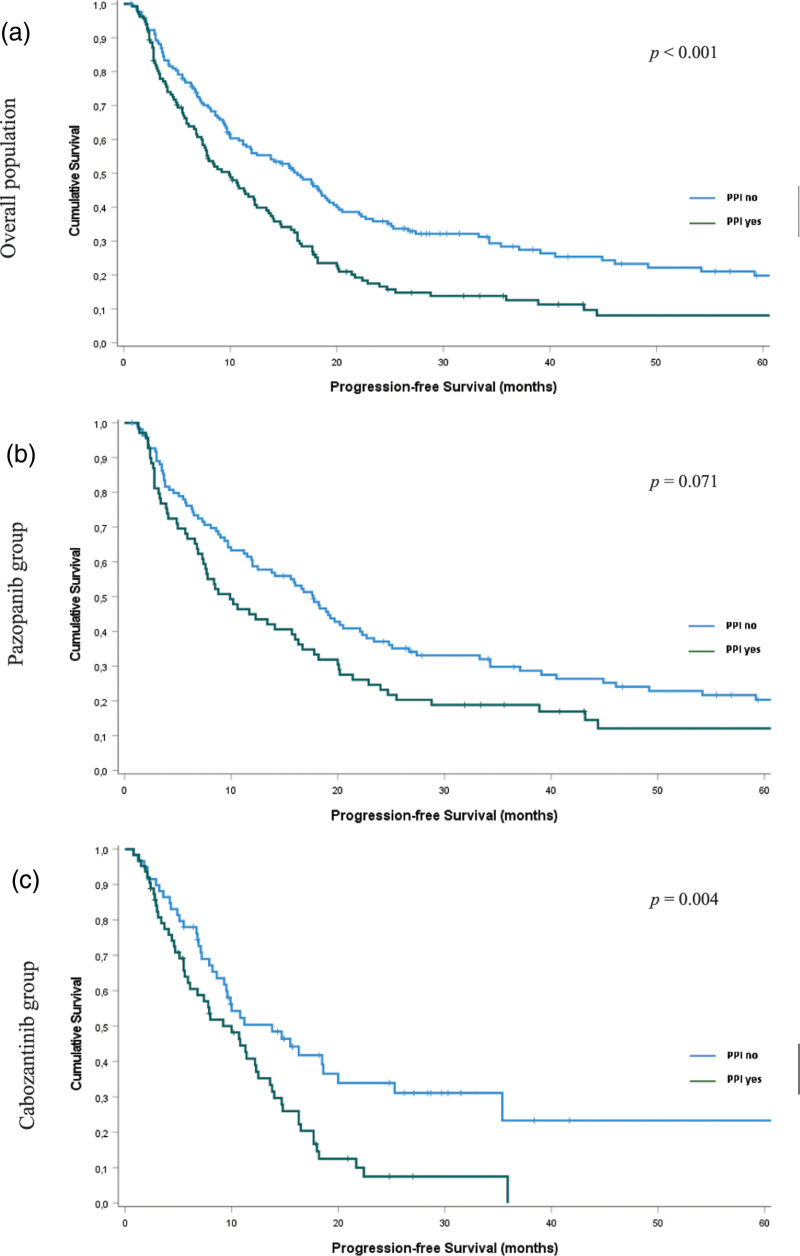
Progression-free survival according to proton-pump inhibitors use: (a) overall population (*n* = 301); (b) pazopanib group (*n* = 179); and (c) cabozantinib group (*n* = 122).

The median follow-up was 46.7 months (95% CI, 35.5–57.9). Median PFS (mPFS) in the overall population was 12.3 months (95% CI, 9.7–14.9). There were 128 events, and mPFS was 16.3 months (95% CI, 12.1–20.5) in patients not taking a PPI and 113 events, with mPFS of 9.9 months (95% CI, 6.9–12.8), in patients taking PPIs (Table 2 and Fig. 1a; *P* < 0.001).

**Table 2:** Univariate and multivariate analyses for progression-free survival and overall survival in the overall population

Covariates	PFS				OS			
	Univariate analyses		Multivariate analyses		Univariate analyses		Multivariate analyses	
	Months (95% CI)	*P*	HR	*P*	Months (95% CI)	*P*	HR	*P*
PPI use								
No	16.3 (12.1–20.5)	<0.001	1.60 (1.22–2.11)	**<0.001**	30.6 (20.4–40.8)	<0.001	1.51 (1.09–2.08)	**0.013**
Yes	9.9 (7.0–12.8)				18.4 (14.7–22.1)			
Age								
<70 years	14.8 (11.8–18.5)	0.047	1.43 (1.09–1.89)	**0.011**	29.3 (23.4–35.2)	0.006	1.87 (1.36–2.58)	**<0.001**
≥70 years	10.0 (7.8–12.2)				22.0 (17.4–26.6)			
IMDC score								
Good	14.1 (9.4–18.8)	<0.001	1.50 (1.19–1.88)	**<0.001**	46.2 (29.2–63.2)	<0.001	2.27 (1.74–2.96)	**<0.001**
Intermediate	14.7 (11.5–17.8)				23.6 (18.3–28.9)			
Poor	4.2 (2.9–5.4)				7.1 (3.3–10.9)			
NLR								
<3	16.0 (10.2–21.7)	0.002	1.45 (1.10–1.92)	**0.008**	33.1 (25.1–41.1)	<0.001	1.48 (1.08–2.04)	**0.016**
≥3	8.8 (5.9–11.7)				18.2 (15.0–21.4)			
ECOG PS								
0	16.0 (13.3–18.7)	<0.001	1.09 (0.85–1.40)	0.487	30.7 (24.5–36.9)	<0.001	1.07 (0.80–1.42)	0.634
1	8.6 (6.9–10.3)				18.4 (14.7–22.1)			
2–3	5.9 (2.0–9.8)				9.5 (3.6–15.4)			
Bone metastases								
No	13.8 (9.6–17.9)	0.007	1.26 (0.95–1.68)	0.102	27.7 (20.6–34.7)	0.014	1.34 (0.97–1.87)	0.077
Yes	11.4 (8.9–13.9)				20.0 (12.8–27.2)			
Node metastases								
No	15.5 (15.4–18.6)	0.051	1.26 (0.94–1.67)	0.111	29.5 (22.9–36.1)	0.004	1.46 (1.06–2.00)	**0.022**
Yes	10.6 (8.2–13.0)				19.7 (14.8–24.6)			

Bold fonts represent statistically significant values (P < 0.05).

ECOG PS, Eastern Cooperative Oncology Group Performance Status; HR, hazard ratio; IMDC, International mRCC Database Consortium criteria; NLR, neutrophil-to-lynphocyte ratio; OS, overall survival; PFS, progression-free survival; PPI, proton-pump inhibitors.

Patients treated with pazopanib had mPFS of 14.1 months (95% CI, 10.4–17.7). In this group, we observed mPFS of 17.7 months (95% CI, 14.5–20.9) in patients who did not use a PPI, whereas patients taking PPIs had mPFS of 9.9 months (95% CI, 5.4–14.4) (Table 3 and Fig. 1b; *P* = 0.071).

**Table 3:** Univariate and multivariate analyses for progression-free survival and overall survival in the pazopanib group

Covariates	PFS				OS			
	Univariate analyses		Multivariate analyses		Univariate analyses		Multivariate analyses	
	Months (95% CI)	*P*	HR	*P*	Months (95% CI)	*P*	HR	*P*
PPI use								
No	17.7 (14.5–20.9)	0.071	1.49 (1.04–2.12)	**0.029**	38.5 (26.7–50.3)	0.188	1.33 (0.89–2.01)	0.158
Yes	9.9 (5.4–14.4)				25.5 (18.5–32.5)			
Age								
<70 years	18.2 (13.9–22.4)	0.047	1.63 (1.12–2.37)	**0.010**	49.2 (26.1–72,3)	0.001	2.38 (1.54–3.70)	**<0.001**
≥70 years	11.9 (8.1–15.7)				25.8 (18.2–33.4)			
IMDC score								
Good	16.7 (10.9–22.5)	<0.001	1.62 (1.20–2.18)	**0.002**	50.7 (37.7–63.7)	<0.001	2.57 (1.82–3.64)	**<0.001**
Intermediate	16.3 (11.2–21.4)				27.8 (17.2–38.4)			
Poor	3.7 (1.4–6.0)				9.2 (6.1–12.3)			
NLR								
<3	19.3 (10.3–28.3)	0.032	1.40 (0.98–1.99)	0.064	42.7 (20.2–65.2)	0.022	1.43 (0.96–2.15)	0.077
≥3	12.0 (4.5–19.5)				25.5 (14.4–36.6)			
ECOG PS								
0	17.6 (14.7–20.5)	0.037	1.15 (0.82–1.61)	0.414	38.5 (29.9–47.0)	0.002	1.05 (0.72–1.54)	0.779
1	8.8 (4.7–12.8)				24.3 (16.7–31.9)			
2–3	6.8 (0.0–18.3)				11.1 (2.8–19.4)			
Bone metastases								
No	16.0 (11.1–20.9)	0.222	1.15 (0.78–1.68)	0.470	35.6 (23.7–47.5)	0.104	1.28 (0.83–1.98)	0.250
Yes	13.4 (10.4–17.8)				25.5 (17.2–33.8)			
Node metastases								
No	15.9 (12.1–19.7)	0.328	1.13 (0.76–1.66)	0.540	35.6 (24.9–46.3)	0.056	1.25 (0.81–1.91)	0.305
Yes	11.2 (6.8–15.5)				22.2 (15.1–29.3)			

Bold fonts represent statistically significant values (P < 0.05).

ECOG PS, Eastern Cooperative Oncology Group Performance Status; HR, hazard ratio; IMDC, International mRCC Database Consortium criteria; NLR, neutrophil-to-lynphocyte ratio; OS, overall survival; PFS, progression-free survival; PPI, proton-pump inhibitors.

Patients treated with cabozantinib had mPFS of 10.8 months (95% CI, 8.2–13.1). In this group, we observed mPFS of 13.8 (95% CI, 7.6–19.9) in patients who did not use PPI, whereas patients taking PPI had mPFS of 10.0 months (95% CI, 6.4–13.5) (Table 4 and Fig. 1c; *P* = 0.004).

**Table 4:** Univariate and multivariate analyses for progression-free survival and overall survival in the cabozantinib group

Covariates	PFS				OS			
	Univariate analyses		Multivariate analyses		Univariate analyses		Multivariate analyses	
	Months (95% CI)	*P*	HR	*P*	Months (95% CI)	*P*	HR	*P*
PPI use								
No	13.8 (7.6–19.9)	0.004	1.64 (1.05–2.57)	**0.004**	26.9 (20.1–33.6)	<0.001	1.92 (1.13–3.25)	**0.015**
Yes	10.0 (6.4–13.5)				15.2 (13.4–16.9)			
Age								
<70 years	12.3 (9.1–15.5)	0.137	1.29 (0.83–2.01)	0.253	18.8 (14.8–22.9)	0.130	1.33 (0.76–2.32)	0.315
*>*70 years	9.2 (6.1–12.3)				16.3 (9.5–23.1)			
IMDC score								
Good	12.5 (8.9–16.1)	0.002	1.34 (0.90–1.98)	0.144	28.4 (22.8–33.9)	<0.001	1.94 (1.25–3.01)	**0.003**
Intermediate	12.2 (7.9–16.5)				16.7 (13.4–20.1)			
Poor	4.4 (3.1–5.7)				6.3 (3.9–8.7)			
NLR								
<3	14.7 (9.9–19.5)	0.026	1.56 (0.99–2.46)	0.054	24.9 (21.4–28.4)	0.024	1.38 (0.80–2.36)	0.238
*>*3	7.4 (3.5–11.2)				15.2 (13.7–16.7)			
ECOG PS								
0	13.8 (10.1–17.5)	0.017	1.10 (0.70–1.71)	0.671	26.1 (17.1–35.1)	0.002	1.50 (0.99–2.25)	0.051
1	8.6 (6.5–10.7)				15.2 (9.8–20.6)			
2–3	3.6 (0.0–8.0)				7.3 (0.0–15.9)			
Bone metastases								
No	11.2 (6.8–15.6)	0.027	1.40 (0.88–2.23)	0.151	18.4 (9.9–26.9)	0.626	0.86 (0.49–1.52)	0.619
Yes	9.9 (6.3–13.5)				16.7 (12.7–20.7)			
Node metastases								
No	12.3 (5.8–18.7)	0.208	1.29 (0.79–2.12)	0.303	18.8 (11.3–26.3)	0.244	1.34 (0.76–2.38)	0.308
Yes	10.0 (7.0–12.9)				16.3 (12.6–20.0)			

Bold fonts represent statistically significant values (P < 0.05).

ECOG PS, Eastern Cooperative Oncology Group Performance Status; HR, hazard ratio; IMDC, International mRCC Database Consortium criteria; NLR, neutrophil-to-lynphocyte ratio; OS, overall survival; PFS, progression-free survival; PPI, proton-pump inhibitors.

#### Overall survival

**Fig. 2:**
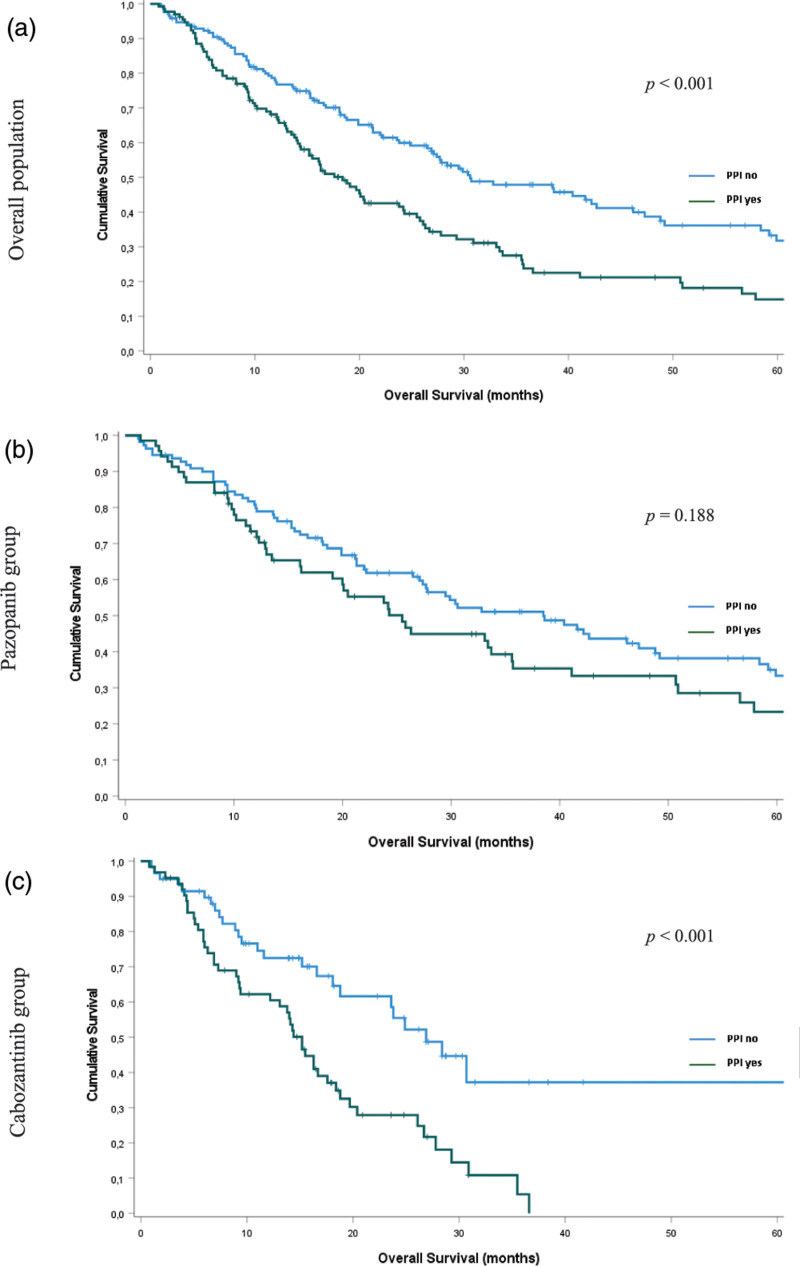
Overall survival according to proton-pump inhibitors use: (a) overall population (*n* = 301); (b) pazopanib group (*n* = 179); and (c) cabozantinib group (*n* = 122).

The overall population’s median OS (mOS) was 25.8 months (95% CI, 21.3–30.3). There were 95 events and mOS of 30.6 months (95% CI, 20.4–40.8) in patients not taking a PPI and 95 events with mOS of 18.4 months (95% CI, 14.7–22.1) in patients treated with PPIs (Table 2 and Fig. 2a; *P* < 0.001).

Patients treated with pazopanib had mOS of 30.6 months (95% CI, 22.1–39.1). In the pazopanib group, we observed an mOS of 38.5 months (95% CI, 26.7–50.3) in patients who did not use a PPI, whereas patients taking PPIs had an mOS of 25.5 months (95% CI, 18.5–32.5) (Table 3 and Fig. 2b; *P* = 0.188).

Patients treated with cabozantinib had an mOS of 18.1 months (95% CI, 14.7–21.5). In the cabozantinib group, we observed an mOS of 26.9 months (95% CI, 20.1–33.6) in patients who did not use a PPI, whereas patients treated with PPI had an mOS of 15.2 months (95% CI, 13.4–16.9) (Table 4 and Fig. 2c; *P* < 0.001).

#### Objective response

In the overall population, 66 (22%) patients were nonresponders and 233 (77%) were responders. Seven patients (2%) obtained CR, 126 (42%) PR, 100 (33%) SD, 56 (19%) PD as best response; 12 patient (4%) were NE for response, 10 of whom due to rapid progression of disease, and two for other reasons. There were 28 nonresponders (17%) and 140 responders (83%) in patients without PPIs compared with 38 (30%) and 93 (71%) in patients taking PPIs, respectively (*P* = 0.012).

In the pazopanib group, the percentage of responders was 77%. Six patients (3%) obtained CR, 77 (43%) PR, 55 (31%) SD, 33 (18%) PD as best response; eight patients (4%) were NE for response. There were 19 nonresponders (17%) and 90 responders (83%) among patients without PPIs compared with 21 (30%) and 48 (70%) among patients taking PPIs, respectively (*P* = 0.064).

In the cabozantinib group, the percentage of responders was 78%. One patient (1%) obtained CR, 49 (40%) PR, 45 (37%) SD, 23 (19%) PD as best response; four patients (3%) were NE for response. There were nine nonresponders (15%) and 50 responders (85%) among patients not taking PPIs compared with 17 (27%), and 45 (73%) among patients taking PPIs, respectively (*P* = 0.124).

#### Toxicity and compliance

Data about toxicity and compliance are summarized in Table 5. Eighty-seven (29%) patients developed grade 3 (G3) or grade 4 (G4) toxicity during treatment with TKIs. There were 52 events (17%) in patients not taking PPIs compared with 35 events (12%) in patients using PPIs (*P* = 0.443). In the pazopanib group, there were 36 events (20%) in patients not taking PPIs compared with 15 events (8%) in patients using PPIs (*P* = 0.128). In the cabozantinib group, there were 16 events (13%) in patients not taking PPIs compared with 20 events (16%) in patients using PPIs (*P* = 0.692).

**Table 5:** Toxicity and compliance to tyrosine kinase inhibitor treatment according to proton-pump inhibitors use

	Overall		Pazopanib		Cabozantinib	
G3-G4 toxicity	PPI no	PPI yes	PPI no	PPI yes	PPI no	PPI yes
	17.3%	11.7%	20.1%	8.4%	13.2%	16.5%
	*P* = 0.443		*P* = 0.128		*P* = 0.692	
Reduction of TKI dose	PPI no	PPI yes	PPI no	PPI yes	PPI no	PPI yes
	31.6%	18.6%	33.0%	15.1%	29.5%	23.8%
	***P* = 0.020**		*P* = 0.066		*P* = 0.106	
Temporaneous interruption of TKI	PPI no	PPI yes	PPI no	PPI yes	PPI no	PPI yes
	29.2%	17.9%	31.8%	15.1%	25.4%	22.1%
	*P* = 0.063		*P* = 0.124		*P* = 0.365	
Modified schedule of TKI	PPI no	PPI yes	PPI no	PPI yes	PPI no	PPI yes
	10.0%	4.7%	5.6%	3.4%	16.4%	6.6%
	*P* = 0.100		*P* = 1.000		***P* = 0.009**	

CTCAE, Common Terminology Criteria for Adverse Event; G3/G4 toxicity, Grade 3/Grade 4 adverse events according to CTCAE; PPI, proton-pump inhibitors; TKI, tyrosine kinase inhibitor.

A total of 151 (50%) patients needed a dose reduction of the TKI during the treatment. There were more dose reductions (31% of cases) among those patients not taking PPIs compared with patients using PPIs (19%; *P* = 0.020), both in the overall population and in the pazopanib (33% vs. 15%; *P* = 0.066) and cabozantinib group (30% vs. 24%; *P* = 0.106).

A total of 142 (47%) patients needed a temporary interruption of the TKI due to toxicity and/or subjective nontolerability. There were more treatment interruptions (29% of cases) among patients not taking PPIs compared with patients using PPIs (18%; *P* = 0.063), both in overall population and in the pazopanib (32% vs. 15%; *P* = 0.124) and cabozantinib groups (25% vs. 22%; *P* = 0.365).

Overall, for 44 (15%) patients, a TKI schedule modification was required, due to toxicity and/or subjective nontolerability. There were more patients (10%) for whom the schedule of TKI was modified among those not taking PPIs compared with patients taking PPIs (5%; *P* = 0.100), both in the overall population and in the pazopanib (6% vs. 3%; *P* = 1.000) and cabozantinib groups (16% vs. 7%; *P* = 0.009).

#### Multivariate analyses

In the univariate analyses, age, IMDC score, NLR, ECOG PS, bone, and nodal metastases resulted as factors significantly associated both with PFS and OS (Table 2). In the multivariate analyses adjusted for these covariates, the use of PPI resulted significantly associated with shorter PFS [hazard ratio (HR), 1.60; 95% CI, 1.22–2.11; *P* < 0.001; Table 2]. Similarly, in the multivariate analysis for OS, the use of PPI was significantly associated with poorer outcomes (HR, 1.51; 95% CI, 1.09–2.08; *P* = 0.013; Table 2).

Tables 3 and 4 show the univariate and multivariate analyses performed in the pazopanib and cabozantinib groups, respectively. In the multivariate analysis in the pazopanib group, the use of PPI was significantly associated with shorter PFS (HR, 1.49; 95% CI, 1.04–2.12; *P* = 0.029; Table 3) and nonsignificantly associated with poorer OS (HR, 1.33; 95% CI, 0.89–2.01; *P* = 0.158; Table 3). In the multivariate analysis in the cabozantinib group, the use of PPI was significantly associated both with shorter PFS (HR, 1.64; 95% CI, 1.05–2.57; *P* < 0.004; Table 4) and poorer OS (HR, 1.92; 95% CI, 1.13–3.25; *P* = 0.015; Table 4).

## Discussion

According to our results, the DDI between PPIs and pazopanib or cabozantinib can significantly impact the outcome of patients to the anticancer therapy (in a detrimental direction) and their treatment compliance. The latter effect seems theoretically favorable, but without a return in terms of clinical effectiveness. Considering the previously published data about the issue, these results are far from obvious [13–18].

Indeed, the few published evidence about cabozantinib seemed reassuring about the safety of the concomitant administration, reporting similar areas under the curve (AUCs) and Cmax of the drug administered with or without esomeprazole in a phase I clinical pharmacology study [13]. Previous data about pazopanib were more controversial, reporting a decrease in the pazopanib Cmax and AUC with the combined use of pazopanib and esomeprazole in a DDI study [14]. The European Organisation for Research and Treatment of Cancer published a pooled analysis of single-arm phase II and placebo-controlled phase III studies of patients with advanced soft tissue sarcoma treated with pazopanib (313 patients were eligible), revealing worse PFS and OS among acid-reducing agents (ARA, including PPIs) users versus nonusers [15]. On the other hand, analyzing a population more similar to that of the present study, a prior retrospective work reported no difference in PFS or OS among ARA users versus nonusers in 90 patients with mRCC treated with pazopanib [16]. Such data seems in contrast to those of the present study, with the limitation of a more limited sample size and the inclusion of histamine-2 receptor antagonists besides PPIs. In addition, the impact observed in our population is consistent with the fact that the efficacy of pazopanib is known to be exposure-dependent: a study on Cmin in patients treated with pazopanib showed that a drug Cmin > 20 mg/l was related to better PFS in mRCC, with a similar trend in sarcoma patients [17].

With regards to the issues of toxicity and compliance, likely strictly related, it is known that toxicity of pazopanib is also exposure-dependent: in the VOTRAGE trial, a phase I dose-escalation study of pazopanib in unfit older patients, patients with dose-limiting toxicities were among the highest pazopanib AUC, and those concomitantly receiving PPIs had a higher oral clearance of the drug compared with those without PPIs [18]. Our data about AEs prevalence are not statistically significant, possibly due to numerically limited subgroups. However, we interpret our compliance results, showing better TKI tolerability (i.e. less G3-G4 toxicity, dose or schedule modifications, and treatment interruptions) in the PPI users group, as the direct consequence of lower exposure to TKIs (Table 5).

The main limitations of the present study are the retrospective nature and the lack of data about the type of PPI, duration, and indication for PPI therapy. On the other side of the coin, this possible heterogeneity grants the reliability about DDI likeliness irrespective of the PPI type and indication for use.

The strengths of our study are represented by the multicenter involvement, the adequate median follow-up, and the bivalence concerning the TKI type (cabozantinib or pazopanib) and treatment line. Our results in terms of OS, PFS, and ORR, beyond the statistical significance, are undoubtedly clinically meaningful enough to deserve careful consideration. With the present data, the tip of the balance of the few available evidence about the issue seems to lean towards the actual existence of a DDI between PPIs and these two TKIs.

In conclusion, the evidence provided by the present study suggests that the use of PPIs can modify the pharmacokinetics of pazopanib and cabozantinib, with a possible reduction of absorption of the TKI and a consequent reduction of the drugs' blood concentration. The lower drug exposure can impact the effectiveness of TKI treatment, possibly explaining a portion of the still-high rate of nonresponders among patients with mRCC. This reflection offers the cue for an immediate modification of our clinical practice, avoiding indiscriminate use of PPIs and recommending a careful selection of patients requiring a gastroprotective therapy.

## Acknowledgements

### Conflicts of interest

M.B. received honoraria as a speaker at scientific events by Bristol-Myers Squibb (BMS), Novartis, AstraZeneca, Pierre Fabre, and Pfizer and as a consultant for advisory role by Novartis, BMS, IPSEN, and Pfizer; she also received fees for copyright transfer by Sciclone Pharmaceuticals and research funding by Roche S.p.A., Seqirus UK, Pfizer, Novartis, BMS, Astra Zeneca, and Sanofi Genzyme. S.B. received honoraria as a speaker at scientific events and advisory role by Bristol-Myers Squibb (BMS), Pfizer; MSD, Ipsen, AstraZeneca, and Novartis; he also received research funding from Novartis. For the remaining authors, there are no conflicts of interest.
